# Evaluating Multi-Target Beam Setup Methods for LINAC-Based Stereotactic Treatment of Multiple Brain Metastases with Individual Dose Prescriptions

**DOI:** 10.3390/cancers18081262

**Published:** 2026-04-16

**Authors:** Xander R. Staal, Jaap D. Zindler, Anna L. Petoukhova

**Affiliations:** 1 Department of Medical Physics, Haaglanden Medical Centre, 2262 BA Leidschendam, The Netherlands; x.staal@haaglandenmc.nl; 2 Department of Radiation Oncology, Haaglanden Medical Centre, 2262 BA Leidschendam, The Netherlands; j.zindler@haaglandenmc.nl; 3 Department of Radiation Oncology, HollandPTC, 2629 JH Delft, The Netherlands

**Keywords:** multiple brain metastases, multiple prescription doses, plan quality, multi-target beam setup, LINAC-based, single-isocenter multi target (SIMT)

## Abstract

**Background/Objectives:** LINAC-based, single-isocenter, non-coplanar, stereotactic radiotherapy (SRT) is an effective treatment for patients with multiple brain metastases (BMs). Within such a treatment plan, individual beams can be assigned to treat a subset of the metastases, this is referred to as multi-target beam setup (MTBS). This work evaluates and compares several strategies for MTBS. **Methods:** A planning study was performed, comparing plan quality parameters for plans resulting from several MTBS strategies, including two automated approaches. Each BM was individually prescribed a dose depending on its volume, resulting in treatment plans with multiple prescription doses. Paddick conformity index and Paddick gradient index were adapted to work with multiple prescription doses. **Results:** Given the same number of treatment beams, distributing BMs over treatment beams gave statistically significant improvements over targeting the BMs with all beams by 2% CI, 9% GI, 15% V15Gy, and 10% V5Gy. No statistically significant difference was found between different methods to distribute BMs over treatment beams. Increasing the number of treatment beams gave a further statistically significant improvement in plan quality at the cost of increased treatment time. **Conclusions:** MTBS is an important tool to increase the quality of non-coplanar LINAC-based stereotactic treatment plans for multiple brain metastases with individual dose prescriptions. MTBS can be automated to save planners a significant amount of time. The MTBS algorithm should be restricted in terms of the number of beams it can generate, to limit treatment times.

## Simple Summary

This work studies the effectiveness of multi-target beam setup (MTBS) methods for LINAC-based, non-coplanar, stereotactic treatment of multiple brain metastases (5 to 20 metastases) with individual dose prescriptions. Within such a treatment plan, each beam can be assigned to treat a subset of the metastases, a process referred to as MTBS. The results indicate that performing MTBS leads to significant improvement in plan quality, compared to treating all metastases in each beam. Results also show that several different MTBS strategies can result in similar plan quality. MTBS can be automated, which saves planners a significant amount of time, especially as the number of metastases and beams in the plan increases. To our knowledge, this is the first article that looks at the effectiveness of MTBS for LINAC-based stereotactic treatment of multiple brain metastases with individual dose prescriptions.

## 1. Introduction

Patients with one or more brain metastases (BMs) and a Karnofsky performance status ≥70 are routinely treated with stereotactic radiotherapy (SRT). Use of SRT was initially limited to patients with up to four BMs; however, following favorable clinical evidence [1,2,3,4] and with improved techniques reducing treatment times, SRT has now become feasible for patients with larger numbers of BMs.

SRT for BMs may be delivered using linear accelerators (LINACs) or specialized devices such as Gamma Knife [5] or CyberKnife [6]. Gamma Knife and CyberKnife are known to deliver highly conformal plans with steep dose gradients [7,8], resulting in high-quality plans, especially for small BMs. However, they are time consuming, costing roughly 20 min per lesion. This makes it an inconvenient modality for treating larger numbers of BMs.

LINAC-based SRT allows the treatment of multiple BMs simultaneously from a single isocenter, with the multi-leaf collimator (MLC) shaping the field appropriately for each BM. This single-isocenter multi-target (SIMT) approach reduces treatment times significantly [9,10,11,12,13] and therefore allows the treatment of patients with larger numbers of BMs. Several studies have found this to be a safe method to deliver SRT treatment to patients with multiple metastases [4,9,14,15,16].

When too many BMs are treated simultaneously using a single isocenter, the MLC may no longer be able to shape the field optimally for each target, and plan quality may suffer. This mainly happens when the MLC cannot conform to all BMs without exposing healthy tissue in between, an issue called island blocking [17,18]. An effective strategy for mitigating island blocking is to partition them into several smaller subsets and have separate beams, each targeting one of those subsets [19]. We refer to this as a multi-target beam setup (MTBS). This allows treatment of up to 20 BMs within 30 min, while retaining good plan quality.

This work compares several MTBS strategies for LINAC-based, single isocenter, non-coplanar SRT of multiple brain metastases, including two automated approaches. Individual dose prescriptions were used, where each BM is separately prescribed a dose based on its volume. This generally results in multiple prescription dose levels within a single treatment plan.

## 2. Materials and Methods

### 2.1. Patient Selection

At Haaglanden MC, patients with brain metastases less than 4 cm in diameter and a Karnofsky score of at least 70 are referred for SRT treatment. Ten of these anonymized patients, with 5 to 20 metastases (median = 12), were selected for this treatment planning study.

For each patient, the planning CT (either Brilliance Big Bore, Philips Healthcare, Andover, MA, USA, or SOMATOM go.Open Pro, Siemens Healthineers, Erlangen, Germany), with a slice thickness of 1 mm, was collected. In addition, the original delineations of organs at risk (OARs) and gross tumor volumes (GTVs) were used. The GTVs were originally delineated on the planning CT along with T1-weighted magnetic resonance images (MRIs).

### 2.2. Dose Prescription

In accordance with the Dutch National Platform Radiotherapy and Neuro-Oncology consensus, dose was prescribed to each metastasis individually, based on the volume of its PTV with a 1 mm GTV-PTV margin. Metastases with PTVs of <1 cc were prescribed 1 × 24 Gy, metastases of 1–10 cc were prescribed 1 × 21 Gy, metastases of 10–20 cc were prescribed 1 × 18 Gy, and metastases ≥20 cc were prescribed 1 × 15 Gy. For metastases in or near the brainstem, GTV-PTV margins were reduced to 0 mm, and dose prescriptions were limited to 1 × 16 Gy. Clinical goals are listed in Table 1. OAR constraints are based on Timmerman [20]; no planning organ at risk volumes (PRV) were used.

### 2.3. Treatment Planning

For each patient, five non-coplanar LINAC-based treatment plans were optimized from scratch in RayStation 2024B (RaySearch, Stockholm, Sweden), each with multiple dual-arc VMAT beams at three couch angles (0°, 60°, 300°). For all patients, plans were created with 6 MV flattening filter free (FFF) energy of a Versa HD LINAC (Elekta, Stockholm, Sweden), including a 6D robotic couch (Hexapod couch, Elekta, Stockholm, Sweden). All plans were calculated using the Collapsed Cone algorithm with a dose grid of 1 mm in three directions using the collapsed cone algorithm. Individual PTVs were distributed over the available beams using five separate methods:

1. **null**: Each PTV is assigned to all available beams.
2. **manual**: The PTV distribution was done manually by our radiotherapy technologists (RTTs). Each PTV is assigned to exactly one beam per couch angle. This is typically done in a way that maximizes separation in the cranial–caudal direction between PTVs assigned to any single beam. BM distributions for 0°, 60°, and 300° couch angles are identical.
3. **scripted**: The PTV distribution was done automatically by a script. Each PTV is assigned to exactly one beam per couch angle. The script maximizes separation in the direction perpendicular to the beam-plan between PTVs assigned to any single beam; this may lead to different BM distributions for each couch angle.
4. **RS-A:** RayStation MTBS, using a predefined number of beams per couch angle. This method partitions and distributes BMs in such a way that it explicitly minimizes island blocking.
5. **RS-B:** RayStation MTBS, using an unlimited number of beams per couch angle. Here, the method is also free to choose the number of dual-arc beams per couch angle.

Methods 4–5 use the built-in MTBS algorithm in RayStation 2024B. In methods 1–4, two beams were used per couch angle for patients with 2–14 metastases, while three beams were used per couch angle for patients with ≥15 metastases. After methods 1–3, collimator angles for each beam were optimized with the Smart Angles function in RayStation. In methods 4–5 this was automatically done by the built-in MTBS algorithm.

### 2.4. Plan Quality Assessment

For each plan, several parameters were extracted, including the volume of the healthy brain (brain-GTV) receiving 5 Gy (V5Gy) and 12 Gy (V12Gy), a conformity index (CI) and gradient index (GI) adapted from Paddick [21,22], and the number of monitor units (MU) used.

#### 2.4.1. Conformity Index for Multiple Prescription Doses

The Paddick conformity index was originally defined for a single prescription dose. In order to apply the concept to our plans containing multiple prescription doses, we modified the calculation as follows:

(1) $$CI=\frac{{V\left[\underset{i}{⋃}\left({PTV}_{i}\cap {PID}_{i,R}\right)\right]}^{2}}{V\left[\underset{i}{⋃}\left({PTV}_{i}\right)\right]\times V\left[\underset{i}{⋃}\left({PID}_{i,R}\right)\right]}$$

Here ${PTV}_{i}$ denotes the combined region of PTVs that were prescribed the ith dose. ${PID}_{i,R}$ represents the region of the isodose corresponding to the ith prescribed dose, lying within R mm of ${PTV}_{i}$. The ∪ and ∩ operators represent the union and intersection of regions, respectively, and V[∙] returns the volume of a region. This formulation effectively calculates the fraction of the total PTV volume receiving its prescribed dose, multiplied by the fraction of the local prescription isodose volumes confined within the PTVs. Note that for a single PTV and/or a single prescription dose with a sufficiently large R, this CI will be identical to the original Paddick conformity index.

This adapted CI includes a range parameter R, which limits the calculation to the region within R mm around each PTV. An optimal R is large enough to ensure that this region completely encompasses the contribution of each PTV to its prescription isodose, but small enough to exclude contributions from nearby PTVs with higher prescription doses. Based on a sensitivity analysis performed with the dose distributions in this study, a value of R = 8 mm was found to be optimal. The 8 mm value represents the point where CI remained the most stable when plotted against R.

#### 2.4.2. Gradient Index for Multiple Prescription Doses

The GI was calculated as:

(2) $$GI=\frac{V\left[\underset{i}{⋃}\left({PID}_{i}\right)\right]}{V\left[\underset{i}{⋃}\left({HPID}_{i}\right)\right]}=\frac{V\left[{PID}_{low}\right]}{V\left[{HPID}_{low}\right]},$$

where ${PID}_{i}$ represents the isodose region for the ith prescribed dose, while ${HPID}_{i}$ is the isodose region for half of the ith prescribed. Physics dictates that higher dose regions are always enveloped in lower dose regions, so for the calculation, we only need to consider the isodose regions pertaining to the lowest prescribed dose.

### 2.5. Plan Validation

All plans were exported to VeriQA version 2.1.20 (PTW, Freiburg, Germany) for secondary dose calculation using the Monte Carlo algorithm (SciMoCa). This secondary dose check software was commissioned based on custom beam models of an Elekta Versa HD accelerator. Dose differences were evaluated within the External contour using a gamma criterion with 3% absolute global dose difference and 1 mm distance-to-agreement. Additionally, relative mean dose differences were evaluated within the combined PTV contour (PTV_tot).

Moreover, absolute film measurements were performed with GafChromic EBT3 films (International Specialty Products, Wayne, NJ, USA) for three patients, with dose differences analyzed using a gamma criterion with 5% absolute global dose difference and 1 mm distance-to-agreement. After irradiation, the films were left to rest for 24 h before being scanned in the center of an EPSON V750 PRO color scanner (Daiwa Kogyo Ltd., Nagano, Japan), using 48-bit RGB mode and maintaining the same scanning direction. The analysis was performed using DoseLab 4.11 [23,24]. A detailed description of this procedure is available elsewhere [25].

### 2.6. Statistical Analysis

Plan quality parameters from the plans with manual MTBS were used as benchmarks. Plan quality parameters from the plans using the other MTBS methods were compared to the benchmark using the Wilcoxon signed-rank test (two-sided, *p* ≤ 0.05). All analyses were performed in SPSS 29.

## 3. Results

### 3.1. Dose Distributions

**Figure 1:**
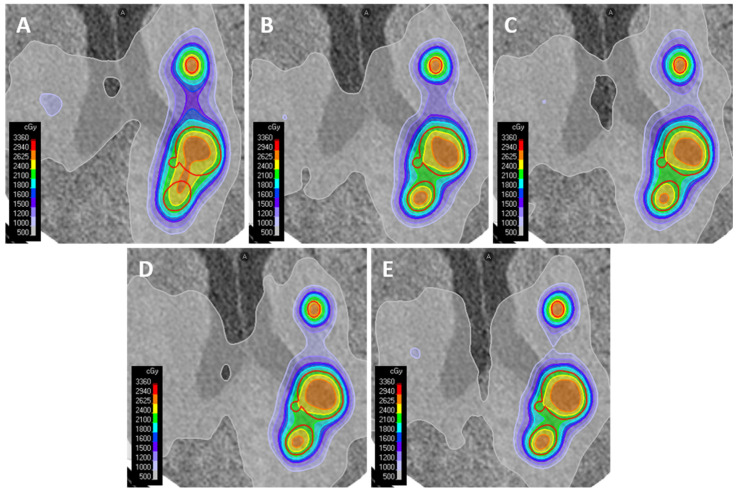
Example dose distributions from the plans made for patient 2, with 6 metastases. (**A**) Plan made with the *null* MTBS method, using 6 beams. (**B**) Plan made with the *manual* MTBS method, using 6 beams. (**C**) Plan made with the *scripted* MTBS method, using 6 beams. (**D**) Plan made with the *RS-A* MTBS method, using 6 beams. (**E**) Plan made with the *RS-B* MTBS method, freely choosing 9 beams. Anterior is shown as (**A**) in the figures.

All plans achieved at least a 99% coverage of PTVs located outside of the brainstem while respecting OAR constraints. Coverage of PTVs located inside the brainstem was allowed to be slightly lower to respect the dose constraint for the brainstem. On visual inspection of the dose distributions (see example in Figure 1), differences were observed in dose bridging between metastases.

### 3.2. Plan Quality

Based on the collected plan quality parameters (Table 2) and corresponding statistical analysis (Table 3), plans using the *null* MTBS method show a statistically significant deterioration in plan quality compared to the *manual* method. Meanwhile, plans using the *scripted* and *RS-A* MTBS methods show no statistically significant change in plan quality compared to the *manual* method. This implies that, when using a fixed number of treatment beams, treating only a subset of the BMs with each beam improves plan quality compared to treating all BMs with each beam. The exact strategy to distribute BMs over beams appears to be less important; the three strategies described in this work (*manual*, *scripted*, *RS-A*) result in similar plan quality.

Plans using the *RS-B* MTBS method show a statistically significant improvement over the *manual* method. The number of treatment beams chosen by the RayStation algorithm in the *RS-B* method was always larger than the fixed number used in the *manual* method. This shows that increasing the number of treatment beams improves plan quality, at the cost of additional treatment time.

**Figure 2:**
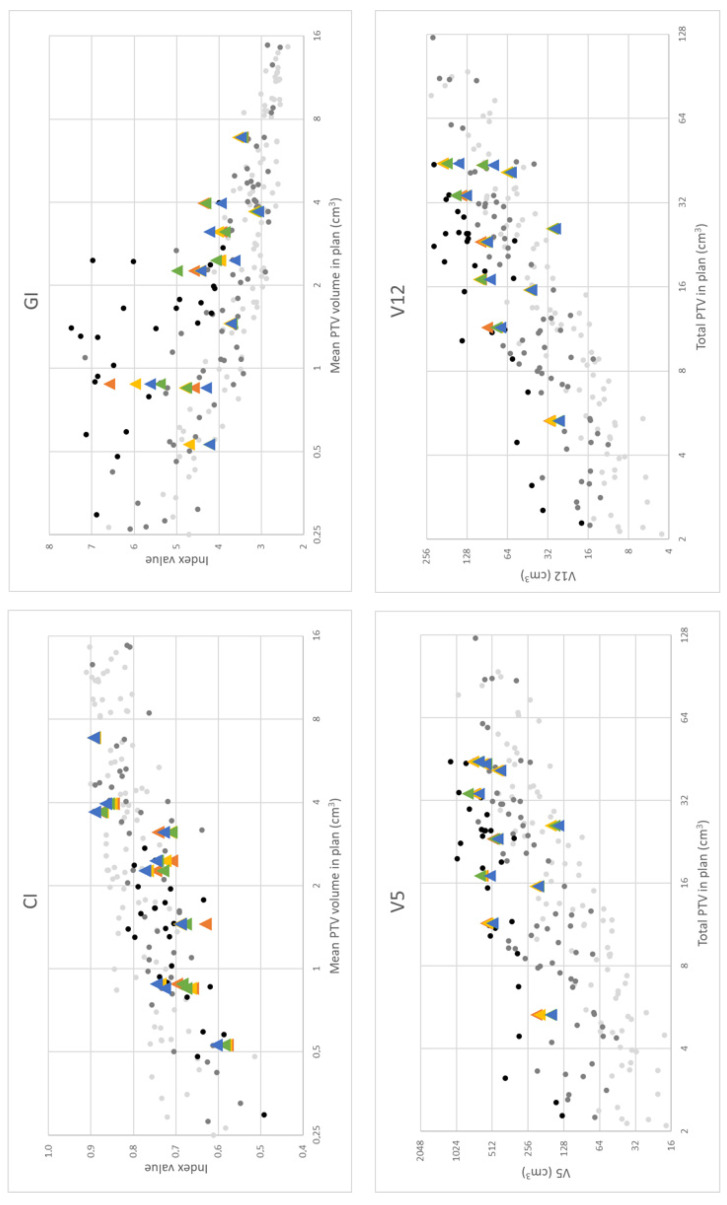
Collected plan quality parameters for plans using MTBS compared to historical data from the clinic. Light gray dots: historical plans with 2–4 BMs. Dark gray dots: historical plans with 5–10 BMs. Black dots: historical plans with 11–20 BMs. Orange triangles: *manual* MTBS method. Yellow triangles: *scripted* MTBS method. Green triangles: *RS-A*. Blue triangles: *RS-B*.

Figure 2 shows the plan quality parameters collected for plans using MTBS in this study and compares them to historical data from our clinic. The plans made in this study fall within the bandwidth of clinical plans. Note that the parameters generally deteriorate for plans with larger amounts of PTVs. CI and GI also deteriorate for smaller PTVs.

The use of multiple prescription doses was only recently introduced in our clinic. Before, a single prescription dose was used for all BMs in a plan based on the largest PTV. The CI and GI for those older plans, therefore, were identical to the original indices defined by Paddick.

### 3.3. Calculation Times

Runtimes of the beam setup algorithms are shown in Table 2. These times represent the time taken to partition the BMs in a plan, then distribute them over the available beams and optimize the collimator angles. Times for the manual beam setup are based on estimations given by experienced RTTs. Note that there is little difference between RS-A and RS-B in terms of runtime, and that the times for these methods increase rapidly from about 18 metastases.

### 3.4. Film Measurements

Film measurements were performed for patients 5, 8, and 9. Table 2 shows gamma pass rates for these measurements. Actual delivery times for treatment plans using the *manual*, *scripted*, and *RS-A* methods were all below 25 min; delivery times for treatment plans using the *RS-B* method were up to 37 min.

## 4. Discussion

LINAC-based single-isocenter non-coplanar SRT is a treatment technique that allows the treatment of up to 20 BMs with individual dose prescriptions within 30 min. MTBS is the act of partitioning the BMs into several smaller subsets, then assigning these to individual treatment beams. This reduces island blocking, improving plan quality. This work evaluates several approaches to MTBS, including a null strategy (where each BM is assigned to all beams), a manual partitioning, a scripted method, and RayStation inbuilt MTBS.

This work was limited to a maximum of 20 BMs. Medically, treating patients with up to 20 BMs using LINAC-based SRT is supported by scientific evidence [26]. So far, to our knowledge, there has been no evidence supporting the use of LINAC-based SRT with more than 20 brain metastases. The total tumor volume and individual tumor volumes of brain metastases are more important factors than the number of metastases in determining local tumor control and the probability of radionecrosis. Clinically, allowing more than 20 metastases is feasible. However, since we generally see plan quality deteriorate with an increasing number of metastases, this should be compensated for by adding additional beams at some point, which might increase treatment times beyond 30 min.

Our results align well with work by Sundström et al. [27], who also show that different algorithms for partitioning metastases result in very similar plan quality when given the same number of beams, and that plan quality increases with a larger number of beams. However, our evaluation uses clinical patients, with multiple prescription doses per plan, and includes secondary dose checks and film dosimetry.

We consistently observed deteriorated gamma pass rates in VeriQA for the RS-B method. This likely has to do with the beams in the RS-B plan being more strongly modulated than in the other plans, using more monitor units to deliver the same dose to the target. Small differences in the MLC-model between RayStation and VeriQA then have a larger impact. This means that the RS-B plans are more likely to be flagged by the secondary dose check, even though we do not observe the same effect in our film measurements. Note that this could impact clinical adoption of the RS-B approach if secondary dose calculation is used as the primary method for plan validation. A deterioration in gamma pass rates would lead to reduced trust in the secondary dose engine and an increased need for dosimetry, which can cost a significant amount of time.

When left unrestricted, the RayStation MTBS algorithm creates many beams, as seen in the results for RS-B. This significantly improves the plan quality parameters shown in this work, at the cost of increased treatment time. We expect that there will be diminishing returns as the number of beams is increased, but this requires more investigation and is outside the scope of this work.

An optimal number of beams should reflect a compromise between plan quality and treatment time. This remains a complex and subjective topic, which should include the prognosis and condition of the patient, and does not have a one-size-fits-all solution. An important practical limit to treatment times in our case is how long a patient can manage to lie still in a mask, which we estimate to be somewhere in the 30–45 min range for most patients.

Another approach to improve plan quality for cases where targets are widely dispersed or when steep dose gradients are most critical is the use of multi-isocenter setups [28,29]. This does come at the cost of increased treatment time. In such an approach, the metastases are distributed over isocenters according to their position in the brain. Teixeira et al. [29] showed that a dual-isocenter technique improved target conformity and reduced low-dose brain exposure compared with a single-isocenter approach, achieving dosimetric performance that closely approximates Gamma Knife using a zero-millimeter GTV–PTV margin. While the patients in our study do include tumor topologies where a multi-isocenter approach could be favorable, we only used single-isocenter setups as is standard practice in our institute [16]. We note that the use of MTBS could potentially also be beneficial in a multi-isocenter setup.

We proposed a modification to the Paddick conformity index and Paddick gradient index to accommodate multiple targets with different prescription doses and show that these parameters allow comparisons with historical data from single-prescription dose plans. The magnitude of the conformity index and gradient index was found to be dependent on the total PTV for studied patients, as for historical patients. A lower CI was observed for smaller total PTVs, suggesting that achieving a conformal treatment plan is more challenging when multiple small target volumes (<1 cc) are present, particularly with a multileaf collimator (MLC) leaf width of 5 mm, as in the Agility MLC system. This limitation has been previously reported [13,30].

For most plans in this study, automated MTBS (scripted, RS-A, RS-B) methods are significantly faster than performing a manual beam setup. However, when the number of metastases in a plan exceeds 18, calculation times for the RayStation MTBS algorithm increase rapidly, and for more than 20 metastases, the calculation time will likely be too long for practical use. This rapid increase in processing time is related to the combinatorial nature of the problem.

## 5. Conclusions

MTBS is an important part of setting up LINAC-based single isocenter non-coplanar stereotactic treatment plans for multiple brain metastases with individual dose prescriptions. When using a fixed number of beams, we expect MTBS to give a significant boost in plan quality compared to treating all BMs with each treatment beam. However, the exact strategy for performing MTBS seems to be less important, with different strategies giving similar results.

MTBS can be automated, as shown here through scripting or by using RayStations built-in method. This will save planners a significant amount of time on beam setup, especially as the number of metastases and beams in the plan increases.

Our modified conformity index and gradient index can be used for treatment plans with multiple targets with separate prescription doses. The resulting values can be directly compared to the Paddick conformity index and the Paddick gradients index calculated on plans with single prescription doses.

## Figures and Tables

**Table 1:** Clinical goals used during planning.

ROI	Description
PTVs	At least 99% volume at 100% of the prescription dose
PTVs	At least 125% of the prescription dose at 1% volume
PTVs	At most 140% of the prescription dose at 0% volume
Brainstem-PTV’s	At most 1600 cGy at 0.03 cc volume
Cochlea L/R	At most 900 cGy at 0.03 cc volume
OpticChiasm	At most 1000 cGy at 0.03 cc volume
OptNrv L/R	At most 1000 cGy at 0.03 cc volume

**Table 2:** Results for all patients. The best performing plans for each patient/result combination are shaded in green, and the second best are shaded in yellow.

	Patient	1	2	3	4	5	6	7	8	9	10
Number of PTVs		5	6	7	10	11	13	15	16	18	20
Total PTV volume [cc]		15.6	41.1	25.9	5.3	43.5	11.4	33.9	23.2	44.2	17.0
Number of beams	Null	6	6	6	6	6	6	9	9	9	9
	Manual	6	6	6	6	6	6	6	9	9	9
	Scripted	6	6	6	6	6	6	6	9	9	9
	RS-A	6	6	6	6	6	6	6	9	9	9
	RS-B	7	9	7	10	10	12	15	14	16	15
MTBS time [m]	Null	1.1	1.2	1.2	1.4	1.4	1.6	2.1	2.1	2.1	2.2
	Manual	10.0	12.0	14.0	20.0	22.0	26.0	30.0	32.0	36.0	40.0
	Scripted	1.4	1.5	1.6	1.9	1.9	2.3	3.3	3.1	3.5	3.7
	RS-A	1.7	1.8	1.7	2.5	2.5	3.2	4.6	5.1	12.5	69.4
	RS-B	1.8	2.2	1.9	2.9	3.1	4.2	5.8	5.9	13.7	70.7
CI	Null	0.69	0.89	0.90	0.62	0.81	0.67	0.75	0.65	0.67	0.58
	Manual	0.74	0.89	0.88	0.58	0.85	0.70	0.75	0.63	0.71	0.66
	Scripted	0.71	0.89	0.87	0.58	0.85	0.74	0.77	0.69	0.73	0.67
	RS-A	0.71	0.89	0.88	0.59	0.86	0.69	0.73	0.68	0.74	0.68
	RS-B	0.73	0.89	0.89	0.61	0.87	0.75	0.77	0.69	0.75	0.73
GI	Null	3.5	3.8	3.4	5.4	4.6	6.7	5.2	4.0	4.2	5.1
	Manual	3.9	3.4	3.1	4.7	4.4	6.6	4.6	3.7	4.0	4.6
	Scripted	4.0	3.6	3.2	4.7	4.3	6.0	4.5	3.8	4.0	4.8
	RS-A	3.9	3.4	3.1	4.2	4.3	5.4	5.0	3.7	4.1	4.8
	RS-B	4.2	3.5	3.1	4.2	4.0	5.6	4.4	3.7	3.6	4.3
V12Gy [cc]	Null	51	74	33	33	122	98	164	112	233	153
	Manual	44	65	30	31	99	91	141	102	196	101
	Scripted	44	65	30	31	99	77	133	96	193	105
	RS-A	43	62	29	27	99	78	156	93	181	102
	RS-B	43	60	28	27	82	72	130	91	149	87
V5Gy [cc]	Null	213	494	187	239	668	631	804	573	815	768
	Manual	214	450	154	218	605	576	726	509	745	617
	Scripted	225	467	166	205	624	559	711	474	737	631
	RS-A	210	441	153	167	572	516	826	491	678	651
	RS-B	211	436	142	164	588	510	665	465	662	538
MU (×1000)	Null	5.0	5.9	6.9	9.1	10.0	9.3	11.0	10.9	11.1	13.7
	Manual	7.9	7.1	10.3	11.5	12.1	10.9	13.5	14.5	13.6	14.9
	Scripted	8.0	7.4	10.1	12.0	11.9	11.4	13.4	13.5	13.8	15.8
	RS-A	8.0	7.0	10.6	11.6	11.0	10.5	13.9	13.8	12.9	14.3
	RS-B	9.6	11.0	11.8	17.7	17.2	18.5	18.3	19.0	19.4	18.8
VeriQA γ 3%/1 mm	Null	100.0	100.0	99.9	99.6	99.9	99.3	99.6	99.4	99.7	92.4
	Manual	99.6	100.0	99.9	97.6	99.2	97.2	98.8	95.9	96.6	88.7
	Scripted	99.2	100.0	98.4	97.4	98.6	96.6	98.3	97.3	98.0	81.6
	RS-A	98.6	100.0	99.5	98.5	99.2	99.7	98.8	96.6	97.2	90.5
	RS-B	97.0	99.6	99.5	87.1	92.3	82.3	96.5	93.2	82.3	80.3
VeriQA mean dose PTV_tot	Null	−0.2	−0.2	2.1	2.7	1.6	0.6	1.1	1.9	0.2	3.4
	Manual	1.7	0.1	2.7	2.4	3.1	1.9	2.1	3.6	2.1	3.8
	Scripted	1.9	−0.1	2.6	5.3	3.1	2.6	2.8	3.4	2.3	4.7
	RS-A	1.4	−0.4	1.9	2.1	1.8	1.4	1.1	2.5	1.2	2.7
	RS-B	2.5	0.7	1.6	5.7	3.4	4.4	2.8	3.4	2.6	4.3
Film γ 5%/1 mm	Manual					98.8			84.6	91.7	
	Scripted					96.4			87.6	97.5	
	RS-A					99.9			86.8	87.2	
	RS-B					99.2			89.7	92.0	

**Table 3:** Pairwise analysis, comparing results from the manual MTBS method with four different MTBS methods as described in Section 2.3. *p*-values as given by the Wilcoxon signed-rank test. Statistically significant values are printed in bold.

Parameter	Null		Scripted		RS-A		RS-B	
	Median % Diff.	*p*	Median % Diff.	*p*	Median % Diff.	*p*	Median % Diff.	*p*
CI	−2.2	0.263	0.8	0.175	0.6	0.401	**4.0**	**0.015**
GI	**8.9**	**0.028**	1.3	0.327	0.0	0.753	−5.4	0.069
V12Gy	**14.9**	**0.005**	0.0	0.138	−3.9	0.086	**−11.9**	**0.005**
V5Gy	**10.2**	**0.008**	0.8	0.859	−3.1	0.263	**−9.7**	**0.008**
MU	**−18.5**	**0.005**	1.4	0.445	−2.6	0.114	**38.9**	**0.005**
VeriQA gamma	**1.5**	**0.012**	0.0	0.260	0.4	0.484	**−5.4**	**0.005**
VeriQA mean dose	**−0.9**	**0.008**	0.5	0.083	**−0.7**	**0.005**	0.9	0.059

## Data Availability

Original data are available by request from the corresponding author.

## Abbreviations

The following abbreviations are used in this manuscript:

SRT	Stereotactic radiotherapy
BM	Brain metastasis
SIMT	Single-isocenter multi-target
MTBS	Multi-target beam setup
MLC	Multi-leaf collimator
OAR	Organ at risk
PTV	Planning target volume
PRV	Planning organ at risk volume
